# EXPLORING AI ELDERS’ CULTURAL PERCEPTIONS OF DISASTERS AND THE ROLE OF GENERATIVITY IN PREPARING FUTURE GENERATIONS

**DOI:** 10.1093/geroni/igad104.0514

**Published:** 2023-12-21

**Authors:** Lena Thompson, Elana Buch, Sato Ashida

**Affiliations:** University of Minnesota, Duluth, Minnesota, United States; University of Iowa, Iowa City, Iowa, United States; University of Iowa College of Public Health, Iowa City, Iowa, United States

## Abstract

Natural and man-made disasters have a great impact on human health, especially for AI Elders. As keepers of cultural and community knowledge, AIAN Elders also serve as guides on how to best support their communities. This study focuses on a Midwestern Tribe’s cultural perceptions of disaster management, specifically being generative, or passing down disaster management knowledge and practices to future generations. In-person semi-structured interviews were conducted with eighteen Elders from the Midwestern Tribe. Recordings were transcribed, de-identified, and coded thematically using an interpretive phenomenological analysis approach. The study received approval from the Tribe’s Department of Emergency Management and the interview guide was reviewed and approved by a Community Advisory Board of six individuals. Elders described past experiences with natural and man-made disasters, including what they did before, during, and after disasters. Elders also explained how people cared for one another, including the importance of interconnectedness with all beings, caring for grandchildren, and the role of the Tribal Government in disaster management. Elders also described how culture shaped disaster management practices, such as traditional practices to follow during a storm, and the important role of Elders in teaching communities what to do when disasters happen. Exploring the role of generativity in passing on disaster-related information is one way to learn how Elders’ knowledge is valued by the community. Findings suggest that Elders can provide important guidance to Tribal governments and public health systems to determine effective disaster management strategies for AIAN communities.

